# Strengthening strategic management approaches to address antimicrobial resistance in global human health: a scoping review

**DOI:** 10.1136/bmjgh-2019-001730

**Published:** 2019-09-11

**Authors:** Raheelah Ahmad, Nina Jiayue Zhu, Andrew J M Leather, Alison Holmes, Ewan Ferlie, the ASPIRES study co-investigators, Raheelah Ahmad, Gabriel Birgand, Enrique Castro-Sánchez, Esmita Charani, Puneet Dhar, Ewan Balfour Ferlie, Mark Ian Hampton, Alison H Holmes [PI], Andy Leather, Mohamed Reda Lebcir, Marc Mendelson, Jules Ndoli Minega, Franco Sassi, Nick Sevdalis, Sanjeev Singh, Carolyn Clare Tarrant

**Affiliations:** 1 NIHR Health Protection Research Unit in Healthcare Associated Infections and Antimicrobial Resistance at Imperial College London, Imperial College London, London, UK; 2 Health Group, Management Department, Imperial College Business School, Imperial College London, London, UK; 3 King’s Centre for Global Health and Health Partnerships, King's College London, London, UK; 4 King’s Business School, King's College London, London, UK

**Keywords:** health policy, review, public health, infections, diseases, disorders, injuries

## Abstract

**Introduction:**

The development and implementation of national strategic plans is a critical component towards successfully addressing antimicrobial resistance (AMR). This study aimed to review the scope and analytical depth of situation analyses conducted to address AMR in human health to inform the development and implementation of national strategic plans.

**Methods:**

A systematic search of the literature was conducted to identify all studies since 2000, that have employed a situation analysis to address AMR. The included studies are analysed against frameworks for strategic analysis, primarily the PESTELI (Political, Economic, Sociological, Technological, Ecological, Legislative, Industry) framework, to understand the depth, scope and utility of current published approaches.

**Results:**

10 studies were included in the final review ranging from single country (6) to regional-level multicountry studies (4). 8 studies carried out documentary review, and 3 of these also included stakeholder interviews. 2 studies were based on expert opinion with no data collection. No study employed the PESTELI framework. Most studies (9) included analysis of the political domain and 1 study included 6 domains of the framework. Technological and industry analyses is a notable gap. Facilitators and inhibitors within the political and legislative domains were the most frequently reported. No facilitators were reported in the economic or industry domains but featured inhibiting factors including: lack of ring-fenced funding for surveillance, perverse financial incentives, cost-shifting to patients; joint-stock drug company ownership complicating regulations.

**Conclusion:**

The PESTELI framework provides further opportunities to combat AMR using a systematic, strategic management approach, rather than a retrospective view. Future analysis of existing quantitative data with interviews of key strategic and operational stakeholders is needed to provide critical insights about where implementation efforts should be focussed, and also how to build contingency at the strategic level for agile responses to macro-level environmental influences.

**Key questions**

**What is already known?**

- National action plans for addressing antimicrobial resistance (AMR) and country-level situation analyses are under way in individual countries and as part of learning networks (Global Antibiotic Resistance Partnership project) but employ different frameworks and approaches which may have local relevance but with temporal validity for policy design and limited transferability to other contexts.

**What are the new findings?**

- Situation analyses for AMR in human heath have not yet employed a strategic management framework which is critical for building contingency at the strategic level for agile responses to macro-level environmental influences.
- Technological and industry analyses is a notable gap.

**What do the new findings imply?**

- Our analysis using a systematic, strategic management approach, rather than a retrospective view shows where important facilitators and inhibitors can be identified and leveraged.

## Introduction

Antimicrobial resistance (AMR) as a serious public health threat is well recognised requiring coordination across governments, country borders, and health and non-health sectors to maintain effectiveness of antimicrobials.^1–3^ While AMR occurs naturally, there are multiple modifiable drivers which accelerate the emergence and spread of resistant pathogens.^4 5^ Wider political, economic, social and epidemiological contextual factors are relevant when thinking about drivers to resistance as well as solutions.^6 7^ These wider influences can facilitate or impede progress because they have direct and indirect effects on individuals (public, patient or healthcare professionals), organisations (healthcare and other sectors) and society as a whole.

Strategies and tools to support national-level interventions include the development and implementation of national action plans (NAPs) for AMR, based on best available evidence.^8^ The evidence base for informing these management strategies requires multidisciplinary approaches including risk assessment,^9 10^ meta-analysis^11^ and cost-effectiveness analysis.^12^

At the global level, in 2011, the WHO initiated a situation analysis of country progress in addressing AMR against four objectives: (1) Improve awareness and understanding of AMR through effective communication, education and training. (2) Strengthen the knowledge and evidence base through surveillance and research. (3) Reduce the incidence of infection through effective sanitation, hygiene and infection prevention measures. (4) Optimise the use of antimicrobial medicines in human and animal health.^13^ Currently only 19 have a NAP with funding sources identified, being implemented and with a defined monitoring and evaluation process in place. A further 43 have plans and processes in place but without funding commitments. Fifty-three countries are in the process of developing plans and 12 have still not initiated this process. With over 60% of countries worldwide at this developmental and planning stage, it is critical that rapid dissemination of learning from those countries that have progressed is shared. The systematic identification of wider influences which could facilitate or threaten progress is needed. Additionally, with use of strategic management approaches, key stakeholders may then work proactively to formulate contingent strategies particularly where wider influences carry high levels of uncertainty.

Country-level situation analyses are under way in individual countries^14^ and as part of learning networks (Global Antibiotic Resistance Partnership—GARP project) all employing different frameworks and approaches which may have local relevance but with temporal validity for policy design and limited transferability to other contexts.^15^

In this paper, therefore, we explore the possible contribution to the analysis of the AMR policy context from a very different literature stream, namely from the discipline of strategic management in management studies. Within this very broad field there are many different schools and approaches.^16 17^ An influential and early school which we access here can be termed the ‘design and planning’ school which seeks to examine the degree of ‘fit’ between an organisation (originally the private firm) and its external environment, using formal methods of rational analysis (rather than more qualitative methods such as visioning or brainstorming). One implication of this design and planning approach is that if the environment is displaying major shifts, then the organisation may need to change too.^18^ A second is that poor fit may well produce poor performance by the organisation.

But how can the very broad notion of the ‘external environment’ be best conceptualised? The design and planning strategic management tradition has produced two important and influential tools of analysis (originally for the private firm,^16^ and later for the public agency^17^), namely SWOT analysis (where Strengths and Weaknesses are internal to the organisation; but Opportunities and Threats come from the external environment), and then the more elaborated PESTEL (and later PESTELI (Political, Economic, Sociological, Technological, Ecological, Legislative, Industry)) framework which draws attention to the following domains: Political factors, Economic influences, Sociological trends, Technological innovations, Ecological factors, Legislative requirements and then Industry analysis.^19^

By performing a systematic search of the literature, this study aims to identify situation analyses conducted in AMR management in different countries and to assess which domains of the PESTELI are included in the extant literature. By doing so, we assess the depth and scope of previous analyses. We also report on the facilitators in addressing AMR identified in this previous research as well as the inhibitors that have been reported.

## Methods

We conducted a literature review to identify situation analyses in addressing AMR.

### Study eligibility

Any study published in English since 2000 that has performed a situation analysis to address AMR was considered in this review, in any country(ies) setting(s). The PICO (Population, Intervention, Comparison and Outcomes)^20^ and SPIDER (sample, phenomenon of interest, design, evaluation, research type)^21^ inclusion and exclusion criteria were applied at the review stages. To capture those studies that do not mention AMR more generally, but rather specific microbes, 13 clinically relevant bacteria (highlighted by Public Health England)^22^ were included in the search (*eg, Escherichia coli*). Studies focussing specifically/solely on drug resistance in tuberculosis, malaria and HIV were excluded. In addition, all of the country-level reports published by the GARP network were included (grey literature).

### Search strategy and information sources

The methods used in this review are in line with the Preferred Reporting Items for Systematic Reviews and Meta-Analyses (PRISMA) guidelines.^23^ The Preferred Reporting Items for Systematic Reviews and Meta-Analyses Protocol (PRISMA-P) checklist was completed as the protocol for literature reviews. The search period was restricted from January 2000 onwards, because there was no policy momentum before this period. The language was limited to English. Studies which did not include AMR in humans were not included. Ovid Medline and Excerpta Medica database (EMBASE), Scopus and EconLit, Healthcare Management Information Consortium (HMIC), PsychInfo, and Institute of Electronical and Electronics Engineers (IEEE) electronic databases were searched. Searches included both controlled vocabulary (predefined subheadings) (eg, Microbials) and text words (eg, gram-negative). The search string used is provided in online supplementary appendix 1.

10.1136/bmjgh-2019-001730.supp1 Supplementary data

### Study selection

Each title and abstract was independently reviewed by two researchers. The full text was independently reviewed by two researchers. Any discrepancies (three) were discussed and re-examined by a third researcher until agreement was reached.

### Assessment of study quality and risks of bias

Formal quality appraisal of individual studies included was not performed, as this would be beyond the aim of this scoping review.^24^ The aim here was to identify gaps in the evidence base and to target topic areas for future research.^25 26^ In addition, use of peer-reviewed publications was used as the proxy for good quality.^24^ In addition, all of the country-level reports published by the GARP network, though grey literature, were also analysed as this network is recognised and cited by the global community for AMR policy design and evaluation.^27^

### Data extraction and analysis

Data extraction was carried out by three researchers (RA, NJZ and EF) and 25% cross-validation between the researchers using a standardised data extraction table (Microsoft Excel), and then mapped against the PESTELI domains. The following information was extracted: study identifiers (title, author list, year of publication, journal, digital object identifier (DOI)), study characteristics (objectives, country/region setting), methods of data collection. Analysis established the scope of the situation analyses in relation to the PESTELI and if any other framework had been used. Facilitators and inhibitors to addressing AMR were mapped against the PESTELI domains. We anticipated descriptive results given the qualitative nature of the studies and hence presented these in text tables and figures.

### Patient and public involvement

This manuscript is part of a wider study, which has one public representative. This representative is a member of the International Advisory Board, and has commented on the early findings of this work in October 2018. This manuscript being a review, did not require further assessment from a patient and public involvement (PPI) perspective. The representative will be actively supporting dissemination of the published work through patient and public networks in South Africa, India and the UK.

## Results

### Included studies

**Figure 1:**
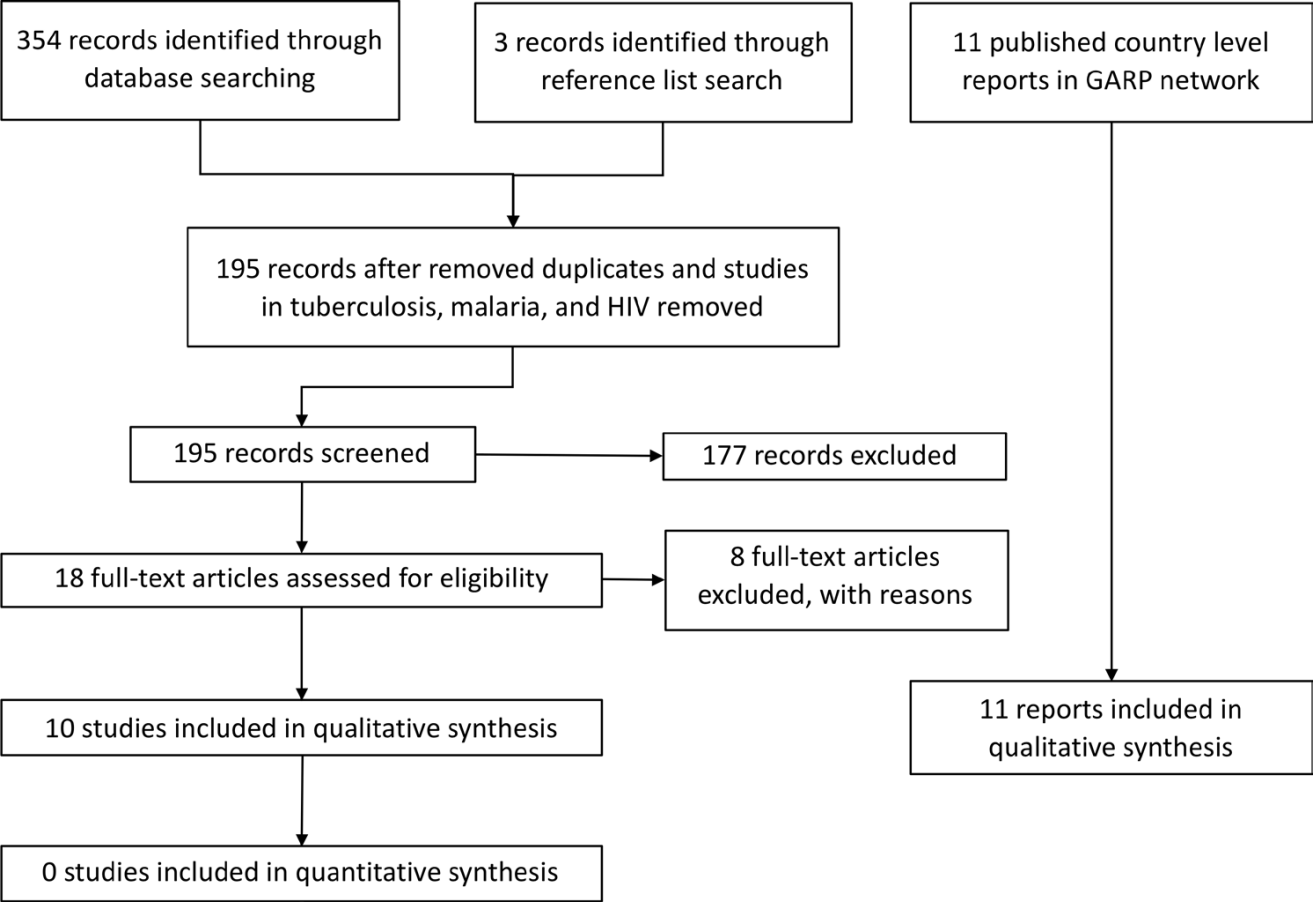
Study flow diagram. GARP, Global Antibiotic Resistance Partnership.

A total of 354 studies was identified from the primary electronic databases with another three from reference list searches. After removal of duplicates and studies in tuberculosis, malaria and HIV, a total of 195 records remained for screening. Eighteen studies were eligible for full-text review and 8 studies were excluded with reasons (2 commentary piece only, 6 organisational-level situation analysis) yielding a total of 10 studies that fit the inclusion criteria. The criteria for study exclusion are provided in online supplementary appendix 2. Figure 1 summarises the flow of literature searching and screening. Additionally, as described above, we included GARP reports published in 11 countries.

### Study characteristics

Of the included studies, six were single-country analysis and four regional-level multicountry studies. In terms of data collection, one study did not collect new data;^28^ one study used primary data collected through interviews with experts;^29^ one study carried out documentary review and interviews;^30^ three studies carried out a documentary review;^31–33^ two used a review of the literature;^34 35^ two studies reviewed literature and collected primary data through interviews with stakeholders.^36 37^

No study employed the PESTELI framework. Most studies (nine) included analysis of the political domain and one study included six domains of the framework. Technological and industry analyses is a notable gap. Only one study employed an established framework (SWOT).^28^ Table 1 presents the domains included in each study.

**Table 1:** Domains of PESTELI (Political, Economic, Sociological, Technological, Ecological, Legislative, Industry) framework covered by the included studies

Study (author/year)	Settings	Data collection	PESTELI framework							
			P	E	S	T	E	L	I	Other frameworks
36	Nepal	Literature review; grey literature; 60 interviews	✓		✓		✓	✓		
34	Tanzania	Literature review; grey literature	✓			✓				SWOT analysis
35	WHO African countries	Literature review	✓			✓				
31	India	Documentary review; case studies	✓	✓	✓			✓		
37	Vietnam	Literature review; stakeholder and expert opinion	✓	✓	✓	✓	✓	✓	✓	
28	Germany	Concept piece—no data		✓		✓	✓	✓		
30	Ghana	Documentary review; four interviews	✓		✓		✓	✓		
32	South-East Asia	Documentary review	✓	✓						
33	South-East Asia	Documentary review	✓	✓						
29	South-East Asia	Concept piece—stakeholder and expert opinion	✓	✓						

SWOT, Strengths, Weaknesses, Opportunities and Threats.

**Figure 2:**
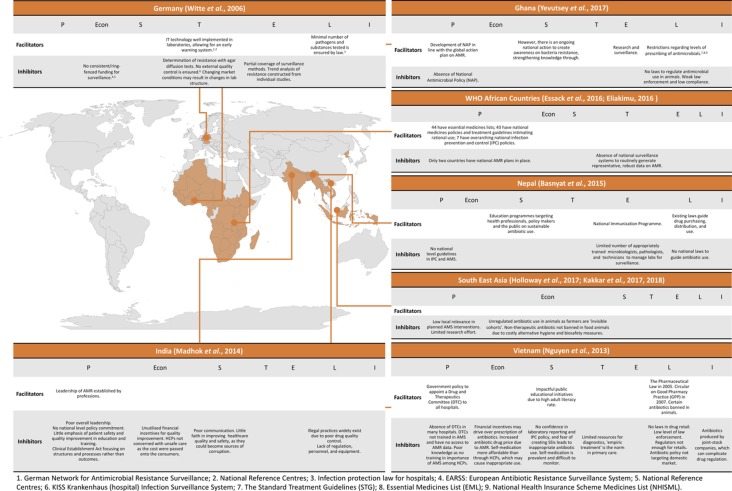
Drivers and inhibitors in addressing antimicrobial resistance (AMR) identified in situation analyses.

Figure 2 provides details the facilitators and inhibitors against the PESTELI framework and the country setting(s).

Facilitators and inhibitors within the political and legislative domains were the most frequently reported. The political domain is featured in each study expect for one.^28^ Facilitators and inhibitors within the political and legislative domains were the most frequently reported. No facilitators were reported in the economic or industry domains but featured inhibiting factors.

Economic inhibitors were lack of ring-fenced funding for surveillance,^28^ unregulated non-therapeutic antibiotics in livestock due to costly alternative hygiene and biosafety measures,^29 32 33^ perverse financial incentives,^37^ cost-shifting to patients.^31^ Industry inhibitors were identified in only one study and reported as complications in regulation arising from joint-stock drug company ownership.^37^

The sociological domain captured education campaigns for the public and healthcare professionals as facilitators. Low level of confidence and motivation to improve quality among healthcare professionals was cited as an inhibitor^31^ and similarly low confidence in laboratory results was reported.^37^ Culture of self-medication and difficulty in monitoring this is reported on the public side.^37^

Among 15 countries that joined the GARP network, 11 have published a country-level report using situation analysis for AMR. Table 2 presents the main areas of findings included in each GARP country report. The reports have followed a structure to cover key areas such as population demographics, economic and health systems context, burden of diseases in humans and animals, AMR in humans, animals and agriculture, and drug regulation and legislation. There is some variability in terms of which areas are investigated in these situation analyses, but all do examine the burden of diseases and antibiotic use in humans and animals. Estimates of burden of AMR and the influence factors associated with the emergence and spread of AMR are missing in some countries. Except for Pakistan and India, all GARP reports describe the policies for regulating antibiotic drugs. Political context is missing in the analyses of nine countries. The heterogeneity of approaches to analysis, may be explained by the incremental phases of the GARP project. The situation analysis published during the first phase of the GARP network (2008–2011) conducted documentary review to synthesise secondary data from government and healthcare institutes. During the second phase (2012–2014), country-level reports have been produced using information in published literature. During the third phase (2015–current), expert opinion and stakeholder interviews have been included in recent situation analysis.

**Table 2:** Areas included in individual GARP country report

Year	Country	Information sources	Main areas covered in the country-level GARP report									
			Demographics	Politic context	Economic context	Health systems setting	Disease burden		Antibiotic use		Drug regulation and supply chain	Other
							Human	Animal	Human	Animal		
2010	Vietnam	Documentary review			✓	✓	✓	✓	✓	✓	✓	
2011	India	Literature review			✓	✓	✓	✓	✓	✓		Interventions to be considered
2011	Kenya	Documentary review		✓			✓	✓	✓	✓	✓	
2011	South Africa	Documentary review			✓	✓	✓	✓	✓	✓	✓	
2014	Nepal	Literature review	✓				✓	✓	✓	✓	✓	
2015	Mozambique	Literature review, expert opinion			✓	✓	✓	✓	✓	✓	✓	
2015	Tanzania	Documentary review	✓				✓	✓	✓	✓	✓	
2015	Uganda	Literature review, interviews	✓				✓	✓	✓	✓	✓	
2017	Zimbabwe	Literature review, interviews			✓	✓	✓	✓	✓	✓	✓	AMR in agriculture
2018	Bangladesh	Documentary review, literature review			✓	✓	✓	✓	✓	✓	✓	Surveillance, requirements in establishing antimicrobial stewardship (AMS) (human resources, education, investment)
2018	Pakistan	Literature review, interviews		✓	✓		✓	✓	✓	✓		Drug accessibility, AMS and interventions
No report available	Seychelles											
	Laos											
	Namibia											
	Nigeria											

AMR, antimicrobial resistance; GARP, Global Antibiotic Resistance Partnership.

The studies identified are all in low and mid-income countries (LMICs) barring one study.^28^

## Discussion

Our review shows that the PESTELI framework for strategic management has not been used for country-level analysis for addressing AMR. Some of the domains of the framework are included in different studies as reported above (figure 2). Analysis of the technological and industry domains is a notable gap. Facilitators and inhibitors within the political and legislative domains were the most frequently reported. No facilitators were reported in the economic or industry domains but featured inhibiting factors.

Learning from the field of strategic management shows that omitting important wider influences when making strategic decisions for addressing AMR can result in policies unaligned with the local landscape and give rise to unintended adverse consequences.^38^

By using a consistent and comprehensive framework such as the PESTELI framework, important facilitators and inhibitors can be identified and leveraged. Key stakeholders may then work proactively to formulate contingent strategies particularly where wider influences carry high levels of uncertainty. For example, in countries which perform well in the economic domain, the factors in the political domain can still hinder progress; notably, in Japan, five terms of ofﬁce, seven general elections since 2000 and an average length of 1.9 years of office (July 1998 and December 2017) may need reliance on strengthening the other domains for sustainable solutions.^39^

Future analysis of existing quantitative data with interviews of key strategic and operational stakeholders, from each of the domains, is needed to provide critical insights about where implementation efforts should be focussed, and also how to build contingency at the strategic level for agile responses to macro-level environmental influences.

Situation analyses for AMR in human heath have not yet employed a strategic management framework which is critical for building contingency at the strategic level for agile responses to macro-level environmental influences.
