# Supplementary material for: Strengthening strategic management approaches to address antimicrobial resistance in global human health: a scoping review
Source: BMJ Glob Health. 2019 Sep 11;4(5):e001730. doi: 10.1136/bmjgh-2019-001730 (PMC6747904; doi:10.1136/bmjgh-2019-001730)
Supplement: Supplementary data [file bmjgh-2019-001730supp001.pdf]

Appendices

Appendix 1. Search string (Ovid syntax)

situation$ analysis.mp.

strateg$ analysis.mp.

environment$ analysis.mp.

SWOT.mp.

PEST.mp.

PESTEL.mp.

PESTELI.mp.

1 or 2 or 3 or 4 or 5 or 6 or 7

antibiotic$.mp.

antimicrobial.mp.

anti-infective.mp.

multidrug.mp.

multi-drug.mp.

microbial.mp.

microbial-drug.mp.

drug-resistan$.mp.

Drug Resistance, Microbial/

acinetobacter.mp.

acinetobacter/

enterococc$.mp.

enterococcus/

escherichia.mp.

escherichia/

enterobacteriaceae/

enterobacteriaceae.mp.

helicobacter/

helicobacter.mp.

streptococcus/

streptococc$.mp.

staphylococcus/

staphylococc$.mp.

klebsiella/

klebsiella.mp.

pseudomonas/

pseudomonas.mp.

neisseria/

neisseria.mp.

chlamydia/

chlamydia.mp.

clostridi$.mp.

clostridium/

gram-negative.mp.

gram-positive.mp.

susceptib$.mp.

nonsusceptib$.mp.

non-susceptib$.mp.

resistan$.mp.

9 or 10 or 11 or 12 or 13 or 14 or 15 or 16 or 17 or 18 or 19 or 20 or 21 or 22 or 23 or 24 or 25 or 26 or 27 or 28 or 29 or 30 or 31 or 32 or 33 or 34 or 35 or 36 or 37 or 38 or 39 or 40 or 41 or 42 or 43 or 44 or 45 or 46

8 and 47 and 48

limit 49 to humans

limit 49 to yr=2000-2019

limit 49 to English

Appendix 2. Study exclusion criteria

Studies were excluded if 1) focused solely on drug resistance in malaria, tuberculosis, or HIV, 2) discussed drivers and inhibitors of the emergence and spread of AMR on organisational level, and 3) included single data source.
